# Transferred Subspace Learning Based on Non-negative Matrix Factorization for EEG Signal Classification

**DOI:** 10.3389/fnins.2021.647393

**Published:** 2021-03-24

**Authors:** Aimei Dong, Zhigang Li, Qiuyu Zheng

**Affiliations:** School of Computer Science and Technology, Qilu University of Technology (Shandong Academy of Science), Jinan, China

**Keywords:** non-negative factorization, transfer learning, shared hidden subspace, EEG signal, classification

## Abstract

EEG signal classification has been a research hotspot recently. The combination of EEG signal classification with machine learning technology is very popular. Traditional machine leaning methods for EEG signal classification assume that the EEG signals are drawn from the same distribution. However, the assumption is not always satisfied with the practical applications. In practical applications, the training dataset and the testing dataset are from different but related domains. How to make best use of the training dataset knowledge to improve the testing dataset is critical for these circumstances. In this paper, a novel method combining the non-negative matrix factorization technology and the transfer learning (NMF-TL) is proposed for EEG signal classification. Specifically, the shared subspace is extracted from the testing dataset and training dataset using non-negative matrix factorization firstly and then the shared subspace and the original feature space are combined to obtain the final EEG signal classification results. On the one hand, the non-negative matrix factorization can assure to obtain essential information between the testing and the training dataset; on the other hand, the combination of shared subspace and the original feature space can fully use all the signals including the testing and the training dataset. Extensive experiments on Bonn EEG confirmed the effectiveness of the proposed method.

## Introduction

Epilepsy (Talevi et al., 2007) is a chronic disease with sudden abnormal discharge of brain neurons, which leads to transient brain dysfunction. Existing studies (Subasi and Gursoy, 2010) have proved that epileptic seizures are caused by sudden abnormal discharge of brain neurons, and the use of EEG signals can effectively improve the progress of epilepsy line detection and diagnosis in order to facilitate the timely treatment of relevant medical staff. Due to its recurrent characteristics, it brings great inconvenience to patients’ daily life. At present, there are about 50 million epileptic patients in the world; most of them come from developing countries. Meanwhile, there are about 2.4 million new patients every year. Epilepsy can occur in all ages, and about 50% of the patients in the world occur in adolescence or childhood. Compared with normal people, the mortality of epileptic patients has increased by 2–3 times.

It is one of the important means to identify and diagnose epilepsy patients with computer-aided therapy according to pathological information contained in the EEG signals. In the classical epilepsy recognition (Guler and Ubeyli, 2007; Tazllas et al., 2009; Dorai and Ponnambalam, 2010; Iscan et al., 2011; Acharya et al., 2013; Fouad et al., 2015) methods, we usually train a classifier to recognize and diagnose epilepsy based on the existing data. The core steps are feature extraction and classifier training. The quality of feature representation is directly related to the training of classifiers. Therefore, in the classification of EEG signals, many methods are generally used to extract the features of EEG signals, such as principal component analysis (PCA) (Subasi and Gursoy, 2010), Kernel principal component analysis (KPCA) (Patel et al., 2018), and wavelet packet decomposition (WPD) (Ting et al., 2008).

With the wide applications of computer-aided diagnosis technology, more and more methods have been applied to EEG signal detection in recent years, such as support vector machine (SVM) (Temko et al., 2011), linear discriminant analysis (LDA) (Subasi and Gursoy, 2010), empirical mode decomposition (EMD) (Bajaj and Pachori, 2012), and fuzzy system (Aarabi et al., 2009). The common characteristic of these methods is that they usually train classifiers to recognize EEG signals according to the existing labeled data. In such cases, great challenges have always been encountered in the process of EEG signal classification. Firstly, the EEG signal is a highly non-linear and non-stationary signal. It is normal situation that different EEG acquisition equipment, different patients, and even the same patient at a different time have different data with diverse characteristics, which leads to the inapplicability of the training model. Second, the number of EEG signals is always insufficient due to the patient’s body or privacy, which also leads to the problems of robustness and generalization of traditional classification methods in EEG signals detection.

To this end, the transfer learning (Dong and Wang, 2014) method is proposed. Transfer learning is a new machine learning method that uses existing knowledge to solve problems in different but related fields. It relaxes two basic assumptions in traditional machine learning: (1) training samples and new testing samples for learning satisfy the condition of independent and identically distribution; (2) the number of samples in the auxiliary domain is much more than that in the target domain. Its purpose is to improve the performance for the target domain with the aid of the auxiliary domain. For the application of epileptic EEG signal classification, health signals and/or signals during seizures are used for training while the testing samples are the signals during seizure-free intervals.

In this paper, we try to solve the problem of epileptic seizure classification with the framework of transfer learning. It is obvious that EEG signals in different fields contain some shared knowledge independent of the data. We reconstruct the EEG signals of different fields to find the shared hidden features between the auxiliary domain and the target domain. In order to improve the recognition ability of the target domain, we augment the dimension of the data and combine the original data with the obtained shared features.

**FIGURE 1:**
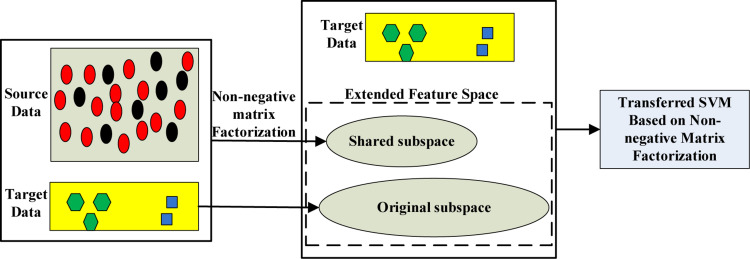
Principle of the proposed transferred SVM based on non-negative matrix factorization.

In summary, we propose a novel method called transferred SVM based on non-negative matrix factorization (Lee and Seung, 1999) (NMF-TL). Specifically, we use a variety of methods to extract the features of EEG signals firstly, and then non-negative matrix factorization is used to extract the shared potential features between the auxiliary domain and the target domain; finally, the augmented dimension is used to train the final classification model in order to improve the discrimination ability of the target domain. The principle of the proposed method is shown in Figure 1.

The rest of the paper is organized as follows. We introduce the feature extraction of EEG signals and the latest transfer learning achievements in Section “Related Work.” In Section “Proposed Method,” the proposed method is formulated in detail. The German EEG data set is used to carry out extensive experiments in Section “Experiments.” Finally, we summarize our method.

## Related Work

In this section, we review the application of feature extraction and transfer learning in EEG signal processing in recent years, as well as the research on non-negative matrix factorization.

### Feature Extraction Methods About EEG Signals

One of the challenges of EEG signal processing is feature extraction. EEG signals have the characteristic of being non-stationary, i.e., the EEG signal is non-linear in nature. At present, there are four EEG signal processing methods: (1) time domain analysis; (2) frequency domain analysis; (3) combination of time and frequency domain; and (4) non-linear method.

Time domain analysis mainly extracts the waveform characteristics of EEG, such as linear prediction (Altunay et al., 2010; Joshi et al., 2014), principal component analysis (Ghosh-Dastidar et al., 2008), independent component analysis (Jung et al., 2001; Viola et al., 2009), and linear discriminant analysis (Jung et al., 2001). Frequency domain analysis uses Fourier transform to extract the frequency characteristics of the EEG signal. Frequency domain analysis can be divided into parametric method and non-parametric method. The non-parametric method extracts frequency domain information of time series. The Welch (Welch, 1967; Polat and Güne, 2007; Faust et al., 2008) method is a typical method. For the non-parametric method’s disadvantage of information loss, the parametric method is proposed. The parametric method mainly includes moving average model, autoregressive (Deryaübeyl and Güler, 2004) model, and autoregressive moving average. Time domain analysis and frequency domain analysis cannot get all the information of the EEG signal separately. So the methods of combining time and frequency domain are proposed, such as wavelet transform (Subasi, 2007) and Hilbert Huang transform (Oweis and Abdulhay, 2011). Non-linear technology can be used to describe the biological system effectively. It is also applicable to EEG signal analysis. Non-linear methods mainly use various parameters of EEG which can describe biological information to extract the features of EEG, such as maximum Lyapunov exponent, correlation dimension, fractal dimension, Hurst index, approximate entropy and sample entropy, and recursive quantitative analysis.

### Non-negative Matrix Factorization

In the process of signal processing, it is an important problem to construct a method that multidimensional data can be better detected. To this end, non-negative matrix factorization (NMF) is proposed; it can extract the potential feature structure of data and reduce the dimension of features.

NMF was proposed by Lee and Sueng (Lee and Seung, 1999). It has obtained great achievements in many fields such as signal processing, biomedical engineering, pattern recognition, computer vision, and image engineering. In recent years, many scholars have improved it from different views. In order to overcome the problem of local and sparse optimization, some scholars (Chen et al., 2001; Li et al., 2001) combine the sparse penalty term with sed as the objective function. However, the local NMF algorithm has poor ability to describe the data. Xu et al. (2003) optimized and proposed a restricted NMF. Wang et al. (2004) added Fisher discriminant information (the difference between intraclass divergence and interclass divergence) into GKLD to form an objective function, and constructed the Fisher NMF algorithm. In order to eliminate the influence of sample uncertainty on data, some weighted NMF (Wang et al., 2006) were also proposed.

For a given domain dataset **X** = [**x**_1_,**x**_2_,⋯,**x**_*N*_] ∈ *ℝ*^*d*×*N*^, *x*_*i*_ = [*x*_*i*1_,*x*_*i*2_,⋯,*x*_*id*_] ∈ *ℝ*^*d*×1^, where *N* is the number of samples and *d* is the number of dimensionality. The goal of non-negative matrix factorization is to find out two non-negative and low-rank matrices: one is coefficient matrix$\text{W}\in {\mathbb{R}}_{+}^{d\times r}$ and the other is base matrix$\text{H}\in {\mathbb{R}}_{+}^{r\times N}$, which satisfy**X**≈**WH**, where*r* < *min*⁡{*d*,*N*}. So the objective function can be defined as follows:

(1) $$\begin{array}{c} \min_{W,H}{||\text{X}\text{ -}\text{WH}||}_{F}^{2}\\ s.t.\text{W}\geq 0,\text{H}\geq 0 \end{array}$$

Lee and Sueng proposed an iterative multiplicative update algorithm and obtained the following update rules:

(2) $${\text{W}}_{i,j}\leftarrow {\text{W}}_{i,j}\,\frac{{\left({\text{XH}}^{T}\right)}_{i,j}}{{\left({\text{WHH}}^{T}\right)}_{i,j}}$$

(3) $${\text{H}}_{i,j}\leftarrow {\text{H}}_{i,j}\,\frac{{\left({\text{W}}^{T}\,\text{X}\right)}_{i,j}}{{\left({\text{W}}^{T}\,\text{WH}\right)}_{i,j}}$$

### Transfer Learning

In the task of EEG signal classification, the traditional machine learning method assumes that all data have the same data distribution. However, due to the non-stationarity of EEG signals, this assumption does not exist, which makes it difficult for traditional methods to achieve good results in practical applications. In order to overcome this problem, transfer learning is put forward.

Transfer learning is proposed to solve small sample problems and personalized problems and has been widely used in BCI classification in recent years. A dual-filter framework (Tu and Sun, 2012) is proposed, which can be used to learn the common knowledge of source domain and target domain. Transfer learning, semi-supervised learning, and TSK fuzzy system are combined (Jiang et al., 2017) to improve the interpretability of transfer learning. In literacy (Yang et al., 2014), with the adoption of the large projection support vector machine, the useful knowledge between the training domain and test domain is learned by calculating the maximum average deviation. In literacy (Raghu et al., 2020), two different classification methods are proposed based on convolutional neural networks: (1) transfer learning by a pre-training network and (2) image feature extraction by a pre-training network and classification by a support vector machine classifier.

## Proposed Method

In this paper, we propose a transfer learning method based on subspace learning. Our method is mainly divided into three steps: the first step is to extract the feature of the EEG signal; the second step is to use non-negative matrix factorization to learn the shared knowledge of the auxiliary domain and target domain; thirdly, the dimension of data is augmented by the combination of the original feature space with the obtained shared feature space. Finally, we use the augmented data space for transfer learning. The principle of the proposed method is shown in Figure 1.

### Notations

(1) Let domain *D* = {**x**_*i*_,*y*_*i*_} ∈ **X**×**Y**:*i* = 1,2,⋯,*n*, where **X** represents the domain sample instance space, *Y* represents the domain sample label space, and {*x*_*i*_,*y*_*i*_} represents an instance in domain *D*.
(2) Let *P*(*D*) = *P*(**X**,**Y**) be the data distribution in domain *D*. There are two domains*D*_*s*_ and *D*_*t*_; if *D*_*s*_≠*D*_*t*_, then *D*_*s*_ and *D*_*t*_ are different domains.
(3) Let $D_{s}=\left\{\left({\text{x}}_{i}^{s},y_{i}^{s}\right)\in {\text{X}}^{s}\times {\text{Y}}^{s},i=1,2,\cdots ,n_{s}\right\}$represent the source domain and $D_{t}=\left\{\left({\text{x}}_{i}^{t},y_{i}^{t}\right)\in {\text{X}}^{t}\times {\text{Y}}^{t},i=1,2,\cdots ,n_{t}\right\}$ represent the target domain, where *n*_*s*_≥*n*_*t*_, the superscript represents the domain, and the subscript represents the index of the sample.

This proposed method is based on the following assumptions: (1) There is only one source domain and one target domain. (2) The data distribution is different but related, and two different domains share a low-dimensional shared hidden subspace through non-negative matrix factorization. (3) The source domain includes a large amount of data and label information, and the target domain includes a small amount of tagged data. The learning task is to make full use of the source domain information to train a classifier with better generalization performance for the target domain.

### Low-Dimensional Shared Hidden Subspace Learning

Given source domain and target domain data **X** = {**X**^*s*^,**X**^*t*^}, where ${\text{X}}^{s}=\left\{{\text{x}}_{1}^{s},{\text{x}}_{2}^{s},\cdots ,{\text{x}}_{n_{s}}^{s}\right\}\in {\mathbb{R}}^{d_{s}\times n_{s}}$ and ${\text{X}}^{t}=\left\{{\text{x}}_{1}^{t},{\text{x}}_{2}^{t},\cdots ,x_{n_{t}}^{t}\right\}\in {\mathbb{R}}^{d_{t}\times n_{t}}$ , *d*_*s*_ and *d*_*t*_ are the numbers of dimensionality in the source domain and target domain, respectively, and*n*_*s*_ and*n*_*t*_ are the numbers of samples in the source domain and target domain, respectively. With the adoption of non-negative matrix factorization, we construct the objective function as Eq. (4):

(4) $$\begin{array}{c} \min_{W^{s},W^{t},H}\alpha^{s}\,{||{\text{X}}^{s}-{\text{W}}^{s}\,\text{H}||}_{F}^{2}+\alpha^{t}\,{||{\text{X}}^{t}-{\text{W}}^{t}\,\text{H}||}_{F}^{2}\\ s.t.\alpha^{s}+\alpha^{t}=1,0<\alpha^{s},\alpha^{t}<1\\ {\text{W}}^{s},{\text{W}}^{t},H>0 \end{array}$$

where**W**^*s*^ ∈ *ℝ*^*d*_*s*_×*r*^ and **W**^*t*^ ∈ *ℝ*^*d*_*t*_×*r*^ are the projection matrices for the source domain and target domain data, respectively, which can map the data from a low-dimensional shared hidden space to the original feature space.*r*is the dimensionality of the low-dimensional shared hidden space and1≤*r*≤*min*⁡{*d*_*s*_,*d*_*t*_}.**H** is the low-dimensional shared hidden space between the source and the target domain. α^*s*^ andα^*t*^ are the weight parameters for the source and target domain and satisfiesα^*s*^ + α^*t*^ = 1. With the adoption of ADMM and literature [27], we obtain the following update rules:

(5) $${\left({\text{W}}^{s}\right)}_{i,j}\leftarrow \frac{{\left({\text{X}}^{s}\,{\text{H}}^{T}\right)}_{i,j}}{{\left({\text{W}}^{s}\,{\text{HH}}^{T}\right)}_{i,j}}\,{\left({\text{W}}^{s}\right)}_{i,j}$$

(6) $${\left({\text{W}}^{t}\right)}_{i,j}\leftarrow \frac{{\left({\text{X}}^{t}\,{\text{H}}^{T}\right)}_{i,j}}{{\left({\text{W}}^{t}\,{\text{HH}}^{T}\right)}_{i,j}}\,{\left({\text{W}}^{t}\right)}_{i,j}$$

(7) $${\left(H\right)}_{i,j}\leftarrow \frac{\alpha^{s}\,{\left({\left({\text{W}}^{s}\right)}^{T}\,{\text{X}}^{s}\right)}_{i,j}+\alpha^{t}\,{\left({\left({\text{W}}^{t}\right)}^{T}\,{\text{X}}^{t}\right)}_{i,j}}{\alpha^{s}\,{\left({\left({\text{W}}^{s}\right)}^{T}\,{\text{W}}^{s}\,\text{H}\right)}_{i,j}+\alpha^{t}\,{\left({\left({\text{W}}^{t}\right)}^{T}\,{\text{W}}^{t}\,\text{H}\right)}_{i,j}}\,{\left(H\right)}_{i,j}$$

Based on the above analysis and derivation, low-dimensional shared hidden subspace learning is obtained. The algorithm description is summarized as shown in Table 1.

**TABLE 1:** The description of the low-dimensional shared hidden subspace learning.

**Algorithm NMF-TL**
1. **Parameters:** Dimensions of shared hidden space *r*, weight parameters for source and target domain α^s^, α^t^
2. **Input:** source domain data $\left\{\left({\text{x}}_{k}^{s},y_{k}^{s}\right)|k=1,2,\cdots ,n_{s}\right\}$, target domain data $\left\{\left({\text{x}}_{k}^{t},y_{k}^{t}\right)|k=1,2,\cdots ,n_{t}\right\}$
3. **Initialization:** set ${\text{W}}_{0}^{s},{\text{W}}_{0}^{t},{\text{H}}_{0}$ satisfying ${\text{X}}^{s}={\text{W}}_{0}^{s}\,{\text{H}}_{0},{\text{X}}^{t}={\text{W}}_{0}^{t}\,{\text{H}}_{0}$, *iter* = 1 set the maximum number *itermax* of iterations and the threshold of error ε
4. **Repeat:**
4-1:update (**H**_*iter*_)_*i,j*_ using Eq.(7)
4-2:update ((**W**_*iter*_)^*s*^)_*i*,*j*_ using Eq. (5)
4-3:update ((**W**_*iter*_)^*t*^)_*i*,*j*_ using Eq. (6)
4-4: *iter* = *iter* + 1
textbfUntil:
∥(**H**_*iter*_)_*i*,*j*_−(**H**_*iter*−1_)_*i*,*j*_∥ < ε or ∥((*W*_*iter*_)_*s*_)*i*,*j*−((*W*_*iter*−1_)^*s*^)_*i*,*j*_∥ < ε
*or* ∥((*W*_*iter*_)*t*)_*i*,*j*_−((*W*_*iter*−1_)^*t*^)_*i*,*j*_∥ < ε *or* (*iter* > *itermax*)
5. **Output:** low dimensional shared hidden space *H* ∈ *ℝ*^*r*×*d*^

### The Process of Training and Testing

After the low-dimensional shared hidden subspace H is obtained, we use H as the shared knowledge between source domain and target domain to transfer information. With the large margin principle, we combine the shared information and SVM conception to learn the final classifier. That is to say, for the training data (source domain data), the classified decision function consists of two parts: the original feature space and the shared hidden space. Specifically, the classified decision function is rewritten based on the classical SVM in the form of Eq. (8):

(8) $$f_{s}\,\left(x\right)={\left({\text{w}}^{s}\right)}^{T}\,{\text{x}}^{s}+{\left({\text{v}}^{s}\right)}^{T}\,{\text{Hx}}^{s}+b^{s}$$

where**w**^*s*^ and **v**^*s*^ represent the classification parameter in the original feature space and shared hidden subspace, respectively. Finally, we use the learned parameters **w**^*s*^, **v**^*s*^, and *b*^*s*^ to classify the testing data (target domain data).

## Experiments

In this section, to evaluate the effectiveness of the proposed method NMF-TL which combines the conception of non-negative matrix factorization, transfer learning, and the large margin principle, we did extensive experiments with EEG signals. All the methods were carried out in MATLAB (R2016b) on a computer with Intel(R) Core (TM) i7-4510U 2.50 GHz CPU and 16GB RAM.

### Dataset and Compared Methods

**FIGURE 2:**
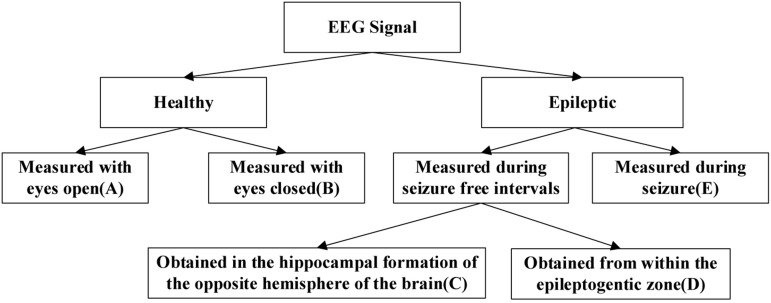
Description of the EEG data.

**FIGURE 3:**
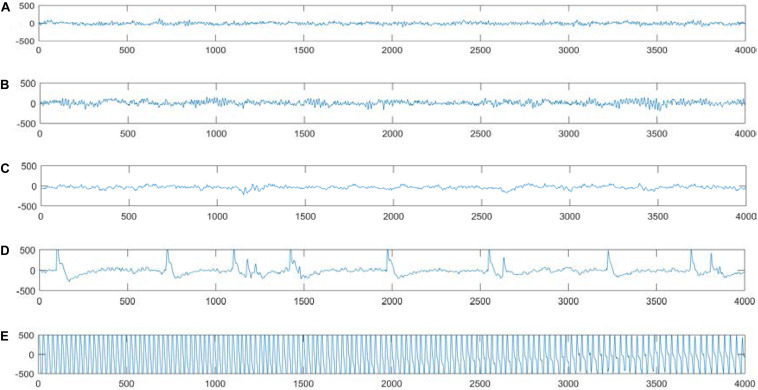
Typical EEG signals in groups **(A–E)**.

The dataset used in the experiments can be publicly downloaded from the web http://www.meb.unibonn.de/epileptologie/science/physik/eegdata.html. The original data contains five groups of data (denoted as A–E), and the details are described in Figure 2. Each group contains 100 single-channel EEG segments of 23.6 s duration. The sampling rate of all datasets was 173.6 Hz. Since there are 100 EEG signals in each group of data, it is not very easy to visualize all their characteristics simultaneously. Figure 3 shows one typical signal in each group to facilitate intuitive observation of the differences in the signals among the five groups of data. The original EEG signals are processed by feature extraction using wavelet packet decomposition (WPD), short-time Fourier transform (SIFT), and kernel principle component analysis (KPCA), and then the EEG signals are used to train and test different classifiers in the experiment.

According to the EEG data described in Figure 2, we designed 10 groups of datasets and each dataset is related with different distributions from two scenarios to compare the performance and effectiveness of the proposed method. In the first scenario, the source domain (i.e., the training dataset) and the target domain (i.e., the testing dataset) are drawn from the identical distribution, while in the second scenario the data distribution is different. The detailed information is summarized in Table 2. Specifically, in scenario 1, dataset 1# is designed for binary classification while dataset 2# is designed for multiclass classification; in scenario 2, datasets 3#–6# are designed for binary classification while datasets 7# and 8# are designed for multiclass classification. For binary classification, we designated the healthy subjects (A or B) as positive class and the epileptic subjects (C, D, or E) as negative class. For multiclass classification, the classification task is to identify different classes according to Figure 2A–E.

**TABLE 2:** The description of the 8 groups of datasets.

**Scenario**	**Datasets**	**Source domain (training dataset)**	**Target domain (testing dataset)**
**Scenario 1: same distribution**	1#	A(75),E(75)	A(25),E(25)
	2#	A(75),B(75),E(75)	A(25),B(25),E(25)

**Scenario 2: different distribution**	3#	A(75),E(75)	A(25),C(25)
	4#	A(75),E(75)	A(25),D(25)
	5#	B(75),E(75)	B(25),C(25)
	6#	B(75),E(75)	B(25),D (25)
	7#	A(75),B(75),E(75)	A(25),B(25),C(25)
	8#	A(75),B(75),E(75)	A(25),B(25),D(25)

A 10-fold cross-validation strategy was used to obtain the final results for scenario 1. For scenario 2, one cross-validation-like strategy was adopted. Specifically, for each dataset in scenario 2, firstly, source data and target data were sampled separately satisfying different distributions to obtain the one classifier; secondly, the source data and the target data are swapped to obtain another classifier. The one-round result is obtained based on the two classifiers. The process is similar to the traditional twofold cross validation strategy. The above procedure was repeated 10 times. For both scenarios, the average result is recorded.

The proposed method is compared with other seven different classification methods, namely, SVM (Guler and Ubeyli, 2007), LDA (Peng and Lu, 2012), DT (Goker et al., 2012), NB (Tazllas et al., 2009), KNN (Cover and Hart, 1967), MTLF (Xu et al., 2017), and LMPROJ (Quanz and Huan, 2009).

### Results and Analysis

The results on classification accuracy of 8 classifiers on 8 different datasets are recorded in Tables 3–5.

**TABLE 3:** Classification accuracy comparison of 8 classifiers on datasets based on WPD feature extraction.

**Method**	**1#**	**2#**	**3#**	**4#**	**5#**	**6#**	**7#**	**8#**
SVM	0.9150	0.6733	0.6842	0.6987	0.9550	0.9650	0.6433	0.6667
LDA	0.9150	0.8600	0.8350	0.8450	0.7850	0.8050	0.8367	0.8300
DT	0.8950	0.7933	0.8500	0.8300	0.9500	0.9300	0.7300	0.7267
NB	0.8700	0.7799	0.5800	0.5600	0.7600	0.7450	0.5799	0.6167
KNN	0.9150	0.8533	0.7650	0.8050	0.9600	0.9500	0.7500	0.7467
MTLF	0.9600	0.8800	0.6950	0.7000	0.9000	0.8850	0.7433	0.7564
LMPROJ	0.8700	0.7767	0.7950	0.8750	0.8000	0.9200	0.6800	0.6700
NMF-TL	0.9700	0.9800	0.9500	0.9500	0.9700	0.9750	0.9699	0.9467

**TABLE 4:** Classification accuracy comparison of 8 classifiers on datasets based on SIFT feature extraction.

**Method**	**1#**	**2#**	**3#**	**4#**	**5#**	**6#**	**7#**	**8#**
SVM	0.9800	0.6908	0.5600	0.5800	0.7150	0.7700	0.7187	0.7033
LDA	0.9900	0.8900	0.5050	0.5650	0.6300	0.6650	0.5700	0.6100
DT	0.9764	0.9300	0.6500	0.7200	0.5600	0.6450	0.6467	0.7067
NB	0.9450	0.9367	0.5800	0.5800	0.5650	0.6400	0.6499	0.6233
KNN	0.9864	0.9367	0.5100	0.5650	0.5100	0.5400	0.6100	0.6333
MTLF	0.9850	0.9833	0.5125	0.5800	0.8450	0.8400	0.6634	0.7067
LMPROJ	0.9800	0.7933	0.6000	0.8750	0.8700	0.8750	0.6700	0.6750
NMF-TL	0.9950	0.9933	0.9700	0.9650	0.9700	0.9650	0.9467	0.9500

**TABLE 5:** Classification accuracy comparison of 8 classifiers on datasets based on KPCA feature extraction.

**Method**	**1#**	**2#**	**3#**	**4#**	**5#**	**6#**	**7#**	**8#**
SVM	0.9300	0.8300	0.5700	0.5645	0.7500	0.7700	0.5933	0.6267
LDA	0.9050	0.5467	0.8900	0.9530	0.9150	0.9150	0.6467	0.6733
DT	0.9800	0.8533	0.8950	0.9725	0.8400	0.8650	0.7767	0.8700
NB	0.8950	0.8149	0.6300	0.7900	0.7900	0.7550	0.6367	0.6700
KNN	0.9400	0.7767	0.8450	0.8950	0.8850	0.9050	0.7400	0.7467
MTLF	0.9350	0.9400	0.7750	0.8500	0.7650	0.8150	0.8199	0.8400
LMPROJ	0.9550	0.9233	0.7717	0.7700	0.8900	0.8400	0.8633	0.8700
NMF-TL	0.9870	0.9500	0.9650	0.9800	0.9800	0.9600	0.9600	0.9767

In Table 3, we give the comparison results of the proposed method and other compared methods based on WPD feature extraction. It can be seen that our method is obviously better than other results. In the results of A/E, B/C, and B/D classification, our method has little improvement effect compared with other methods, with an increase of about 6%. However, in other group classifications, our method improves the effect obviously, and it improves the accuracy by more than 10%. This also proves that our method can better learn the shared knowledge between source domain and target domain.

In the STFT feature classification results shown in Table 4, we can see that our method has achieved good results in other groups of experiments except the A/E group. This is because A/E classification is a traditional binary classification and the proposed method has not demonstrated the superiority over other compared method. For the A/B/E group experiment, our method has improved the accuracy of about 9% compared with the other non-transfer learning methods and improved about 5% compared with the other two transfer learning methods. In all the other group experiments, the proposed method achieved a better range of results.

From Table 5, we can see that our method has improved by about 4% compared with other methods in the A/E group classification. In other groups of experiments, our method has improved about 12% accuracy compared with several baseline methods and also improved about 5% accuracy compared with the other two transfer learning methods.

In summary, from Tables 3–5, we can draw the following conclusion:

(1) For the traditional scenario, i.e., the scenario where the training dataset and the testing dataset are drawn from the same distribution, the proposed method could not demonstrate the superiority over other compared methods, especially for binary classification tasks.
(2) For the transfer learning scenario, the i.e., scenario where the training dataset and the testing dataset are drawn from different but related domains, the transfer learning methods can achieve better results compared with the non-transfer learning methods. The results display that the transfer learning method can exert the positive transfer ability to the best advantage.
(3) For the transfer learning scenario, i.e., the scenario where the training dataset and the testing dataset are drawn from different but related domains, the proposed method shows better performance compared with the other two transfer learning methods. These results show that the proposed method can not only find the shared hidden knowledge but also find the potential relationship between the source domain and the target domain.

**FIGURE 4:**
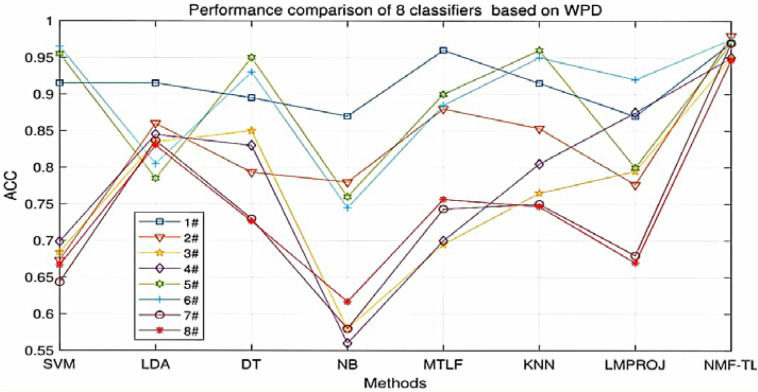
Classification accuracy comparison of 8 classifiers on datasets based on WPD feature extraction.

**FIGURE 5:**
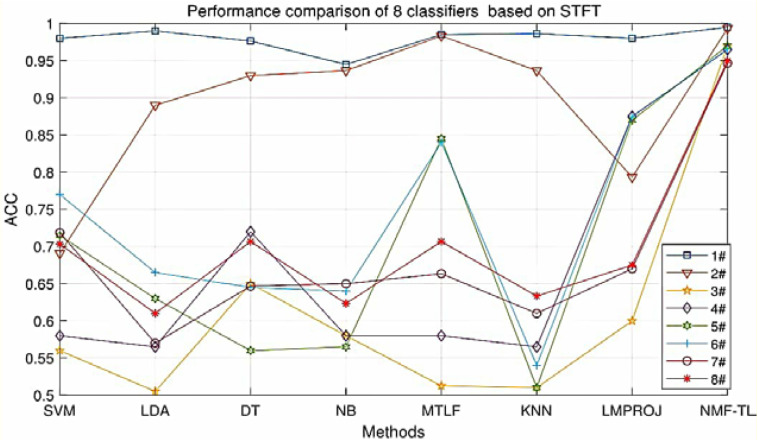
Classification accuracy comparison of 8 classifiers on datasets based on SIFT feature extraction.

**FIGURE 6:**
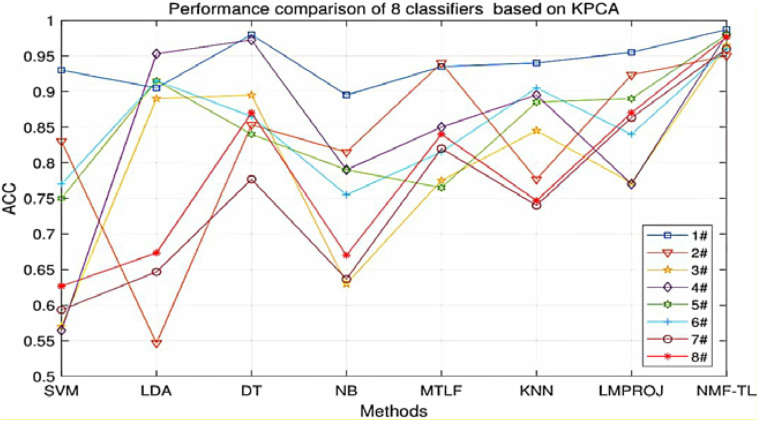
Classification accuracy comparison of 8 classifiers on datasets based on KPCA feature extraction.

At the same time, in order to make our experimental results more visual, we give a broken line chart of the accuracy of our experimental results as shown in Figures 4–6. From Figures 4–6, we can clearly see that our experimental method is obviously better than other experiments in accuracy, and our experimental method has greatly improved the experimental accuracy compared with other methods.

Besides the classification accuracy, we also performed experiments with measurements of F1 score and Recall.

In Table 6, we compare the F1_score results of our method with other methods based on WPD feature extraction. It can be seen that our method is superior to other methods except the B/C and B/D dataset. In the comparison between A/C and A/D, our method only improves about 0.25%. But in other comparison results, the F1_score of this method is improved by about 7%.The proposed method can find the potential relationship between the source and the target domain by non-negative matrix factorization and balance the performance between accuracy and recall. LDA has also achieved good results in this experiment, which shows that LDA classification has good generalization ability.

**TABLE 6:** F1_score of 8 classifiers on datasets based on WPD feature extraction.

**Method**	**1#**	**2#**	**3#**	**4#**	**5#**	**6#**	**7#**	**8#**
SVM	0.9364	0.8033	0.9784	0.9685	0.9562	0.9587	0.6877	0.6954
LDA	0.9600	0.9100	0.9000	0.9000	0.9700	0.9700	0.9100	0.9100
DT	0.9000	0.7600	0.8700	0.8700	0.9500	0.9500	0.7600	0.7600
NB	0.8659	0.8050	0.2092	0.3303	0.8022	0.7922	0.8050	0.7850
KNN	0.9600	0.8550	0.9000	0.9700	0.9900	0.9800	0.8450	0.8450
MTLF	0.9559	0.8850	0.5578	0.5994	0.8888	0.8885	0.8700	0.8700
LMPROJ	0.8678	0.8535	0.8109	0.8725	0.8260	0.9126	0.8025	0.7684
NMF-TL	0.9987	0.9850	0.9800	0.9700	0.9800	0.9800	0.9850	0.9800

The F1_score comparison results of 8 classification methods based on KPCA feature extraction are shown in Table 7. The proposed method has achieved good results except A/E and A/C groups. Compared with other baseline methods, the F1_score of the proposed method in the A/B/E, B/C, and B/D groups increased by about 5%, and that in the A/B/C and A/B/D groups increased by about 15%; compared with the other two transfer learning methods, the F1_score of our method in the A/C and A/D groups increased by about 18%, and that in the B/D, A/B/C, and A/B/D groups increased by about 4.5%.

**TABLE 7:** F1_score of 8 classifiers on datasets based on PCA feature extraction.

**Method**	**1#**	**2#**	**3#**	**4#**	**5#**	**6#**	**7#**	**8#**
SVM	0.9014	0.8840	0.9572	0.9685	0.6503	0.6462	0.5972	0.6211
LDA	0.9100	0.5100	0.9000	0.9100	0.8700	0.8700	0.5300	0.5300
DT	0.9890	0.8450	0.9767	0.9823	0.8500	0.8500	0.8100	0.8200
NB	0.7930	0.8150	0.4302	0.7403	0.7759	0.7562	0.8152	0.4850
KNN	0.9800	0.7700	0.9300	0.9400	0.9100	0.9000	0.7700	0.7700
MTLF	0.9341	0.9200	0.7046	0.8131	0.8374	0.8274	0.9200	0.9200
LMPROJ	0.9566	0.9434	0.7806	0.8066	0.8984	0.8376	0.9037	0.9078
NMF-TL	0.9800	0.9450	0.9700	0.9980	0.9800	0.9400	0.9700	0.9700

In Table 8, we show the F1_score comparison of eight classification methods based on STFT feature extraction. It can be seen that compared with the other baseline methods, the proposed method has increased by about 8% in the A/B/E and A/B/D experimental groups, and that in the A/B/C experimental group increased by about 6%; compared with the other two transfer learning methods, it increased by about 66% in the A/C experimental group and in other experimental groups obvious improvement has also been observed.

**TABLE 8:** F1_score of 8 classifiers on datasets based on SIFT feature extraction.

**Method**	**1#**	**2#**	**3#**	**4#**	**5#**	**6#**	**7#**	**8#**
SVM	0.9796	0.8264	0.9796	0.9765	0.8042	0.8218	0.7239	0.7470
LDA	0.9898	0.8500	0.9672	0.9801	0.9600	0.9765	0.8500	0.8500
DT	0.9987	0.9050	0.9253	0.9645	0.9900	0.9900	0.9050	0.9050
NB	0.9667	0.8850	0.4248	0.4247	0.6927	0.6972	0.8850	0.8900
KNN	0.9845	0.9150	0.9632	0.9754	0.9847	0.9667	0.9100	0.9100
MTLF	0.9900	0.9800	0.2720	0.3995	0.6766	0.8626	0.9391	0.9066
LMPROJ	0.9649	0.8661	0.3307	0.8871	0.8803	0.8812	0.8816	0.8016
NMF-TL	0.9920	0.9950	0.9987	0.9600	0.9800	0.9900	0.9700	0.9950

We record the recall results of 8 classification methods based on WPD feature extraction in Table 9. As shown in Table 9, compared with the baseline method, the recall rate of the proposed method in the A/B/C and A/B/D groups increased by about 22%; compared with the two transfer learning methods, the recall rate of our method in the A/C and B/C experimental groups increased by about 4%, and the recall rate in the A/D, B/D, and A/B/C experimental groups increased by about 6.5%.

**TABLE 9:** Recall of 8 classifiers on datasets based on WPD feature extraction.

**Method**	**1#**	**2#**	**3#**	**4#**	**5#**	**6#**	**7#**	**8#**
SVM	0.9600	0.9980	0.9700	0.9600	0.9700	0.9700	0.5100	0.5100
LDA	0.8700	0.7600	0.7700	0.7900	0.6000	0.6400	0.6900	0.6700
DT	0.8900	0.8600	0.8300	0.7900	0.9500	0.9100	0.6700	0.6600
NB	0.8400	0.8581	0.1200	0.2200	0.9600	0.9600	0.6521	0.6674
KNN	0.8700	0.8500	0.6300	0.6400	0.9300	0.9200	0.5600	0.5500
MTLF	0.9700	0.9393	0.5150	0.5300	0.9100	0.9050	0.7815	0.7757
LMPROJ	0.8600	0.9750	0.8700	0.8600	0.9200	0.8400	0.9750	0.9750
NMF-TL	0.9400	0.9700	0.9200	0.9300	0.9600	0.9700	0.9400	0.8800

In Table 10, we can see that the proposed method has achieved good results in terms of recall rate. In the B/C group, the difference is only 1.5% compared with the optimal result. In A/E, the proposed method is 0.87% higher than the optimal value. In the A/B/E, A/C, A/D, and A/B/C groups, the NMF-TL method has achieved the best results. In the B/C group, the proposed method is only 1.5% lower than the optimal value, which indicates that the NMF-TL method is good in this group of experiments.

**TABLE 10:** Recall of 8 classifiers on datasets based on SITF feature extraction.

**Method**	**1#**	**2#**	**3#**	**4#**	**5#**	**6#**	**7#**	**8#**
SVM	0.9800	0.8350	0.8650	0.9650	0.9750	0.9850	0.6300	0.6650
LDA	0.9800	0.9700	0.0400	0.1300	0.2600	0.3300	0.0400	0.1300
DT	0.9900	0.9800	0.3000	0.4400	0.1300	0.3000	0.1300	0.3100
NB	0.9875	0.9548	0.4100	0.4100	0.9750	0.9800	0.6618	0.6594
KNN	0.9900	0.9870	0.0400	0.1300	0.0400	0.0800	0.0400	0.0800
MTLF	0.9850	0.9847	0.2391	0.3000	0.9750	0.9635	0.6588	0.7049
LMPROJ	0.9700	0.9550	0.2000	0.9575	0.9200	0.9125	0.9525	0.9675
NMF-TL	0.9987	0.9900	0.9400	0.9700	0.9600	0.9400	0.9000	0.8600

From Table 11, we can see that except the A/B/E and A/B/C groups, the proposed method has achieved the best results. At the same time, in the A/B/E group the proposed method is only 1% lower than the optimal value and in A/B/C group, the difference is more, which is a decrease by 3%.

**TABLE 11:** Recall of 8 classifiers on datasets based on PCA feature extraction.

**Method**	**1#**	**2#**	**3#**	**4#**	**5#**	**6#**	**7#**	**8#**
SVM	0.9550	0.9650	0.9200	0.9400	0.5600	0.5600	0.4600	0.4600
LDA	0.9000	0.6200	0.8800	0.9600	0.9600	0.9600	0.8800	0.9600
DT	0.9600	0.8700	0.7900	0.9800	0.8300	0.8800	0.7100	0.9700
NB	0.7700	0.8643	0.2800	0.7500	0.7100	0.7100	0.6809	0.8060
KNN	0.9800	0.7700	0.9300	0.9400	0.9100	0.9000	0.7700	0.7700
MTLF	0.9200	0.9898	0.7000	0.7000	0.9500	0.9525	0.7898	0.8534
LMPROJ	0.9600	0.9450	0.9500	0.9550	0.9700	0.9200	0.9600	0.9600
NMF-TL	0.9700	0.9600	0.9900	0.9600	0.9800	0.9800	0.9300	0.9700

In summary, from the recall results shown in Tables 9–11, we can draw the following conclusion:

(1) Recall rate means the probability of being predicted as a positive sample in the actual positive sample. In Table 9, we can clearly see that our method has achieved good results, which also proves that our method rarely has misdiagnosis results in the detection process and improves the accuracy of our diagnosis results.
(2) In the diagnosis of diseases, there will be misdiagnosis. A good detection method can greatly reduce the incidence of misdiagnosis. In this experiment, our method is obviously better than other methods.
(3) The higher the recall rate, the lower the misdiagnosis rate of the correct samples. The lower the misdiagnosis rate in medical diagnosis, the more conducive it is to the relevant practitioners to make judgment as soon as possible. In this group of experiments, our method has achieved good results, which shows that compared with other methods, our algorithm has a lower misdiagnosis rate.

### Friedman and Nemenyi Tests

**FIGURE 7:**
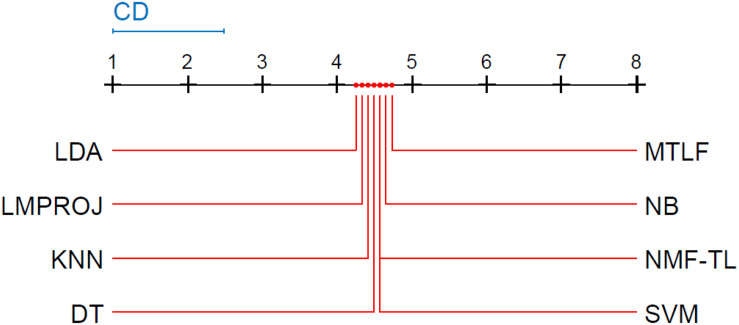
Nemenyi test chart of 8 different methods on datasets based on WPD feature extraction.

**FIGURE 8:**
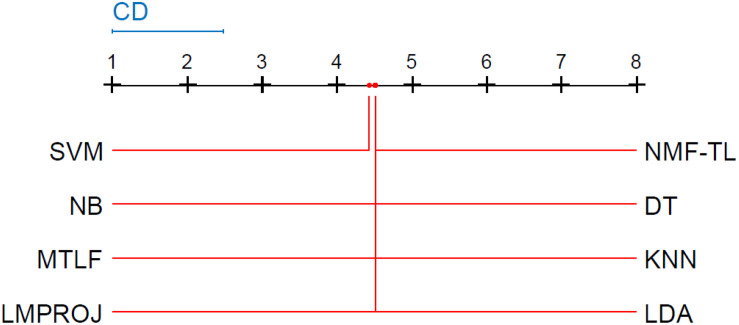
Nemenyi test chart of 8 different methods on datasets based on SIFT feature extraction.

**FIGURE 9:**
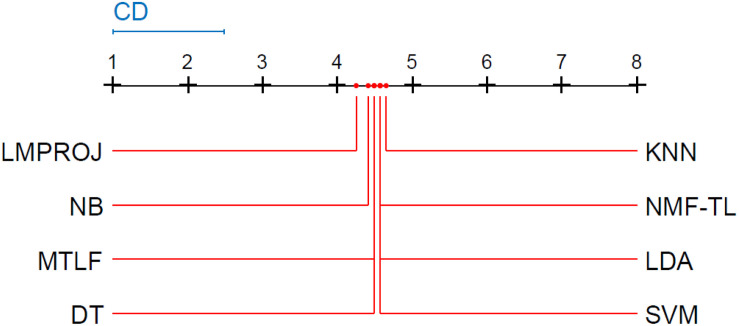
Nemenyi test chart of 8 different methods on datasets based on KPCA feature extraction.

Friedman and Nemenyi tests are used to compare several algorithms on 8 different datasets. The Friedman test can analyze whether there exist obvious differences between all comparison algorithms on multiple data sets. Nemenyi was used to further analyze whether those pairs of algorithms have significant differences. In Tables 12–14, we report Friedman values for each algorithm on 8 datasets with three different feature extraction methods. Figures 7–9 show the Nemenyi test chart for each algorithm on 8 datasets with three different feature extraction methods.

**TABLE 12:** Friedman values for 8 different methods on datasets based on WPD feature extraction.

**Method**	**1#**	**2#**	**3#**	**4#**	**5#**	**6#**	**7#**	**8#**
SVM	1.420	37.81	36.01	36.64	0.4300	1.200	38.14	27.41
LDA	7.128	11.42	1.48	3.528	9.148	3.020	3.045	12.23
DT	0.7375	3.788	0.6325	1.418	0.5150	0.8775	0.2700	1.408
NB	1.818	6.735	25.40	17.75	9.423	11.36	28.11	20.25
KNN	2.260	2.368	3.562	1.470	9.910	1.628	13.25	3.648
MTLF	0.1725	0.4475	12.60	10.80	3.980	5.590	12.16	10.86
LMPROJ	2.250	14.74	1.788	0.3450	5.288	3.245	28.73	30.51
NMF-TL	0.8150	0.3700	2.853	1.178	1.255	0.3225	1.238	1.023

**TABLE 13:** Friedman values for 8 different methods on datasets based on SIFT feature extraction.

**Method**	**1#**	**2#**	**3#**	**4#**	**5#**	**6#**	**7#**	**8#**
SVM	0.430	35.27	36.06	36.64	27.27	11.71	15.88	9.250
LDA	16.50	16.00	14.52	13.55	18.67	21.72	18.12	17.78
DT	8.593	23.98	7.800	12.89	8.280	15.47	14.95	30.32
NB	0.1075	1.283	23.04	25.43	31.86	25.96	25.13	33.13
KNN	16.80	35.85	17.12	27.64	16.65	31.67	15.76	28.85
LMPROJ	1.00	14.00	26.10	4.310	1.010	0.9300	21.73	38.74
MTLF	0.4125	0.085	35.11	28.40	8.670	8.648	33.47	26.43
NMF-TL	0.4300	0.2850	6.195	0.7825	0.5150	3.300	3.865	2.028

**TABLE 14:** Friedman values for 8 different methods on datasets based on KPCA feature extraction.

**Method**	**1#**	**2#**	**3#**	**4#**	**5#**	**6#**	**7#**	**8#**
SVM	14.75	37.46	28.83	7.850	9.773	33.44	8.365	27.28
LDA	1.565	0.9150	2.570	4.422	2.220	0.6975	0.7675	2.165
DT	1.223	1.010	2.085	2.727	2.143	2.800	2.433	4.095
NB	2.213	21.05	5.057	21.55	2.513	1.095	13.15	2.580
KNN	1.165	0.3075	3.162	0.8975	1.445	0.3050	0.2450	4.475
LMPROJ	0.5450	0.4900	11.59	8.845	2.665	5.875	4.033	35.63
MTLF	0.1725	0.3825	12.73	7.200	7.773	5.955	11.04	5.973
NMF-TL	0.3625	0.7600	0.2575	0.6225	0.8150	2.307	0.2150	2.545

From Tables 12–14, we draw the following conclusions.

(1) For WPD feature extraction, it can be seen that the proposed method has achieved good results in several groups. In the experiments of the A/B/E, B/D, and A/B/D groups, our results have won the first place; in the comparison of the A/E, A/C, and B/C groups, ours got the third place; and in the rest of the groups, ours got the second place. We can see that the proposed method has obvious differences with other algorithms, especially with SVM, LDA, NB, and KNN. This is because the traditional classification method is not suitable for transfer learning circumstances which need to find the potential relationship between the source and the target domain.

(2) For SIFT feature extraction, our method got the first place in most of the experiments, the third place in the A/E group, and the second place in the B/D group.
(3) For KPCA feature extraction, our experimental results are almost the same as those of other feature extractions and we also get the best results in many groups, but the results in the A/B/E and B/D groups are not very ideal, and our results are not as good as those of other experiments.

The horizontal line in Figure 7 indicates the size of the average order value. The solid dot on the horizontal line represents the average order value of each corresponding algorithm. The blue line represents the size of the CD value. The red line represents the CD value of each algorithm. The more there are overlapping red lines, the more similar the performance of the two algorithms. From Figure 7, we can see that our method is significantly higher than the critical value CD compared with other methods, and it also shows that our method has a completely different performance from other methods.

From Figure8, we can see that the values of several models are significantly larger than the CD value, which also shows that our method is significantly different from other methods based on SITF feature extraction, and there is no model similar to our experimental model. At the same time, in addition to SVM, other models are similar.

From Figure 9, we can see that compared with other groups of experiments, the *p*-value we obtained in this group of experiments is the largest, which shows that compared with WPD and SIFT feature extraction, there are greater differences in the models of this group of experiments. We can see that the performance of our method is not as good as other methods, such as LMPROJ, NB, MTLF, and DT. This is because our method needs to extract the shared potential features between the source and the target domain, which leads to the performance degradation of our method. In terms of performance, LDA and SVM are most similar to our method.

## Conclusion

In this paper, we proposed new transfer learning methods based on non-negative matrix factorization with the large margin principle for EEG signal classification. Specifically, we first learned the shared hidden subspace data between the source domain and the target domain, then we trained the SVM classifier on the augmented feature space consisting of the original feature space and the shared hidden subspace, and finally we use the learned classifier to classify the new target domain data. Extensive experiments confirmed the effectiveness of the proposed method. As future work, we will evaluate the proposed method on more new datasets, such as the Chinese physiological signal challenge dataset on electrocardiogram classification.

## Data Availability Statement

The dataset analyzed for this study can be found in the Department of Epileptology University of 19 Bonn (http://epileptologie-bonn.de/cms/upload/workgroup/lehnertz/eegdata.html).

## Author Contributions

AD developed the theoretical framework and model in this work and drafted and revised the manuscript. ZL and QZ implemented the algorithm and performed experiments and result analysis.

## Conflict of Interest

The authors declare that the research was conducted in the absence of any commercial or financial relationships that could be construed as a potential conflict of interest.

## References

[B1] Aarabi A. Fazel-Rezai R. Aghakhani Y. (2009). A fuzzy rule-based system for epileptic seizure detection in intracranial EEG. *Clin. Neurophysiol.* 120 1648–1657. 10.1016/j.clinph.2009.07.002 19632891

[B2] Acharya U. R. Sree S. V. Swapna G. Martis R. J. Suri J. S. (2013). Automated EEG analysis of epilepsy: a review. *Knowl. Based Syst.* 45 147–165. 10.1016/j.knosys.2013.02.014

[B3] Altunay S. Telatar Z. Erogul O. (2010). Epileptic EEG detection using the linear prediction error energy. *Expert Syst. Appl.* 37 5661–5665. 10.1016/j.eswa.2010.02.045

[B4] Bajaj V. Pachori R. B. (2012). Classification of seizure and nonseizure EEG signals using empirical mode decomposition. *IEEE Trans. Inform. Technol. Biomed.* 16 1135–1142. 10.1109/titb.2011.2181403 22203720

[B5] Chen X. Gu L. Li S. Z. Zhang H.-J. (2001). “Learning representative local features for face detection,” in *Proceedigs of the IEEE Computer Society Conference on Computer Vision and Pattern Recognition. CVPR 2001* (Kauai, HI: IEEE).

[B6] Cover T. M. Hart P. E. (1967). Nearest neighbor pattern classification. *IEEE Trans. Inform. Theory* 13 21–27.

[B7] Deryaübeyl E. Güler I. (2004). Spectral analysis of internal carotid arterial Doppler signals using FFT. AR, MA, and ARMA methods. *Comput. Biol. Med.* 34 293–306. 10.1016/s0010-4825(03)00060-x 15121001

[B8] Dong A. M. Wang S. T. (2014). A shared latent subspace transfer learning algorithm using SVM. *Acta Automatica Sinica* 40 2276–2287.

[B9] Dorai A. Ponnambalam K. (2010). “Automated epileptic seizure onset detection,” in *Proceedings of the International Conference On Autonomous and Intelligent Systems (AIS), 2010* (Piscataway, NJ: IEEE), 1–4. 10.3233/jifs-200800

[B10] Faust O. Acharya R. U. Allen A. R. Lin C. M. (2008). Analysis of EEG signals during epileptic and alcoholic states using AR modeling techniques. *Irbm* 29 44–52. 10.1016/j.rbmret.2007.11.003

[B11] Fouad M. Amin K. M. El-Bendary N. Hassanien A. E. (2015). “Brain computer interface: a review,” in *Brain-Computer Interfaces. Intelligent Systems Reference Library*, Vol. 74 eds Hassanien A. Azar A. (Cham: Springer), 3–30.

[B12] Ghosh-Dastidar S. Adeli H. Dadmehr N. (2008). Principal component analysis-enhanced cosine radial basis function neural network for robust epilepsy and seizure detection. *IEEE Trans. Biomed. Eng.* 55 512–518. 10.1109/tbme.2007.905490 18269986

[B13] Goker I. Osman I. Ozekes S. Baslo M. B. Ertas M. Ulgen Y. (2012). Classification of junenile myoclonic epilepsy data acquired through scanning electromyography with machine learning algorithms. *J. Med. Syst.* 36 2705–2711. 10.1007/s10916-011-9746-6 21681512

[B14] Guler I. Ubeyli E. D. (2007). Multiclass support vector machines for EEG-signals classification. *IEEE Trans. Inform. Technol. Biomed.* 11 117–126. 10.1109/titb.2006.879600 17390982

[B15] Iscan Z. Dokur Z. Demiralp T. (2011). Classification of electroencephalogram signals with combined time and frequency features. *Expert Syst. Appl.* 38 10499–10505. 10.1016/j.eswa.2011.02.110

[B16] Jiang Y. Wu D. Deng Z. Pengjiang Qian P. Wang J. Wang G. (2017). Seizure classification from EEG signals using transfer learning. Semi-Supervised Learning and TSK Fuzzy System. *IEEE Trans. Neural Syst. Rehabil. Eng.* 25 2270–2284. 10.1109/tnsre.2017.2748388 28880184

[B17] Joshi V. Pachiori R. B. Vijesh A. (2014). Classification of ictal and seizure-free EEG signals using fractional linear prediction. *Biomed. Signal Process. Control* 9 1–5. 10.1016/j.bspc.2013.08.006

[B18] Jung T. P. Makeig S. Mckeown M. J. Bell A. J. Lee T. W. Sejnowski T. J. (2001). Imaging brain dynamics using independent component analysis. *Proc. IEEE Inst. Electr. Electron. Eng.* 89 1107–1122. 10.1109/5.939827 20824156 PMC2932458

[B19] Lee D. D. Seung H. S. (1999). Learning the parts of objects by non-negative matrix factorization. *Nature* 401 788–791. 10.1038/44565 10548103

[B20] Li S. Z. Hou X. W. Zhang H. J. Cheng Q. S. (2001). “Learning spatially localized, parts-based representation,” in *Proceedings of the 2001 IEEE Computer Society Conference on Computer Vision and Pattern Recognition. CVPR 2001* (Kauai, HI: IEEE).

[B21] Oweis R. J. Abdulhay E. W. (2011). Seizure classification in EEG signals utilizing Hilbert–Huang transform. *BioMed. Eng. OnLine* 10:38. 10.1186/1475-925x-10-38 21609459 PMC3116477

[B22] Patel R. Gireesan K. Sengottuvel S. (2018). Decoding non-linearity for effective extraction of the eye-blink artifact pattern from EEG recordings. *Pattern Recognit. Lett.* 139 42–49. S0167865518300291.

[B23] Peng P. Lu B. L. (2012). “Immune clonal algorithm based feature selection for epileptic EEG signal classification,” in *Proceedings of the 11th International Conference on Information Science, Signal Processing and their Applications (ISSPA)* (Montreal, QC: IEEE), 848–853.

[B24] Polat K. Güne S. (2007). Classification of epileptiform EEG using a hybrid system based on decision tree classifier and fast Fourier transform. *Appl. Math. Comput.* 187 1017–1026. 10.1016/j.amc.2006.09.022

[B25] Quanz B. Huan J. (2009). “Large margin transductive transfer learning,” in *Proceedings of the.18th ACM Conference on Information And Knowledge Management* (New York, NY: Association for Computing Machinery), 1327–1336.

[B26] Raghu A. B. S. Sriraam N. Temel Y. Rao S. V. Kubben P. L. (2020). EEG based multi-class seizure type classification using convolutional neural network and transfer learning. *Neural Netw.* 124 202–212. 10.1016/j.neunet.2020.01.017 32018158

[B27] Subasi A. (2007). EEG signal classification using wavelet feature extraction and a mixture of expert model. *Expert Syst. Appl.* 32 1084–1093. 10.1016/j.eswa.2006.02.005

[B28] Subasi A. Gursoy M. I. (2010). EEG signal classification using PCA. ICA, LDA and support vector machines. *Experts Syst. Appl.* 37 8659–8666. 10.1016/j.eswa.2010.06.065

[B29] Talevi A. Cravero M. S. Castro E. A. (2007). Discovery of antivonvulsant activity of abietic acid through application of linear discriminant analysis. *Bioorg. Med. Chem. Lett.* 17 1684–1690. 10.1016/j.bmcl.2006.12.098 17234417

[B30] Tazllas A. T. Tsipouras M. G. Fotiadis D. I. (2009). Epileptic seizure detection in EEGs using time-frequency features. *IEEE Trans. Inf. Technol. Biomed.* 13 703–710. 10.1109/titb.2009.2017939 19304486

[B31] Temko A. Thomas E. Marnane W. Lightbody G. Boylan G. (2011). EEG-based neonatal seizure detection with support vector machines. *Clin. Neurophysiol.* 22 464–473. 10.1016/j.clinph.2010.06.034 20713314 PMC3036797

[B32] Ting W. Guo-Zheng Y. Bang-Hua Y. Hong S. (2008). EEG feature extraction based on wavelet packet decomposition for brain computer interface. *Measurement* 41 618–625. 10.1016/j.measurement.2007.07.007

[B33] Tu W. T. Sun S. L. (2012). A subject transfer framework for EEG classification. *Neurocomputing* 82 109–116. 10.1016/j.neucom.2011.10.024

[B34] Viola F. C. Thorne J. Edmonds B. Schneider T. Eichele T. Debener S. (2009). Semi-automatic identification of independent components representing EEG artifact. *Clin. Neurophysiol.* 48 1470–1480. 10.1016/j.clinph.2009.01.015 19345611

[B35] Wang G. Kossenkov A. V. Ochs M. F. (2006). LS-NMF: a modified non-negative matrix factorization algorithm utilizing uncertainty estimates. *BMC Bioinformatics* 7:175. 10.1186/1471-2105-7-175 16569230 PMC1450309

[B36] Wang Y. Jia Y. Hu C. Turk M. (2004). Fisher non-negative matrix factorization for learning local features. *Res. Gate* 2004 27–30.

[B37] Welch P. D. (1967). The use of fast Fourier transform for the estimation of power spectra: a method based on time averaging over short, modified periodograms. *IEEE Trans. Audio Electroacoust.* 15 70–73. 10.1109/tau.1967.1161901

[B38] Xu B. Lu J. Huang G. (2003). “A constrained non-negative matrix factorization in information retrieval,” in *Proceedings of the IEEE International Conference on Information Reuse and Integration IRI 2003*, Las Vegas, NV: IEEE.

[B39] Xu Y. H. Pan S. J. L. Xiong H. Wu Q. Luo R. Min H. (2017). A unified framework for metric transfer learning. *IEEE Trans. Knowl. Data Eng.* 29 1158–1171.

[B40] Yang C. J. Deng Z. Choi K. S. Jiang Y. Wang S. (2014). Transductive domain adaptive learning for epileptic electroencephalogram recognition. *Artif. Intell. Med.* 62 165–177. 10.1016/j.artmed.2014.10.002 25455561

